# Progress of Diseases of the Throat and Nose

**Published:** 1902-01-04

**Authors:** 


### PROGRESS OF DISEASES OF THE THROAT AND NOSE.

**Sclerotic Hyperplasia.**—An apparently unique case of sclerotic hyperplasia of the pharynx and nasopharynx is recorded by Brown Kelly.[1] The patient, a male aged 34, had an enlargement of the uvula which "hung like a curtain," concealing the posterior pharyngeal wall, and was more than 1¼ inch long; its colour was pale and consistence fibrous. The posterior pharyngeal wall was also thickened in two longitudinal bands, of grey colour and with perfectly smooth surface. Both arytenoid regions in the larynx showed similar smooth, firm thickening. Histologically the tissue was found to consist of dense connective tissue of sparsely cellular character, disposed in denser whorls around the blood vessels. The author considers that the possibility of the disease being rhinoscleroma or syphilis was excluded, and that it was analogous to idiopathic subglottic hypertrophic laryngitis of Bosworth and other authors.

**Adenoids.**—In the course of a discussion on general anæsthesia for operations in the nose and throat Emil Mayer[2] stated that he had reported his experience in 250 cases operated on for adenoids under *ethyl bromide* anæsthesia but had used it at least 500 times, and in only two instances had it been necessary to interrupt the anæsthetic because of unpleasant symptoms. He used a mask which almost excluded the air from the nose and mouth. He claimed that it was harmless in quantities of 10 to 15 grammes, that anæsthesia was quickly induced and lasted from a minute-and-a-half to two-and-a-half minutes, and that it produced no unpleasant after effects. The majority of operators begin by pouring 10 grammes upon a mask and use altogether 30 or 40 grammes. Roaldes[3] said that the enthusiasm he had evinced ten years ago when reporting 200 cases anæsthetised by bromide of ethyl remained almost unabated, for he could say that in over 5,000 cases he had met with unpleasant effects only two or three times. This anæsthetic did not depress the heart like chloroform, and its action was a little less fugitive than that of nitrous oxide. It was most important to use a pure preparation and one that had been preserved in a sealed glass tube.

**Mercurial Lesions.**—Schmithuisen[4] recently described some of the signs of chronic mercurial poisoning of the throat which begin at the base of the tongue, between the tubercles of the mucous glands and the lingual tonsil. The affected parts are characterised at first by dark, smooth spots, which soon become white, and later greyish-yellow. The spots occur almost always before similar appearances in the mouth. Schmithuisen considered the diagnosis very difficult, especially the differential diagnosis from syphilitic papules, but the latter became better, while the mercurial lesions were aggravated by the continued use of mercury.

**Hypertrophy of the Tonsils.** — The removal of chronically enlarged tonsils by enucleation is commended by St. Clair Thomson[5] in certain cases. Two years previously he had removed the embedded tonsilar stumps which had remained after reduction by galvano-cauterisation, and which had continued to cause troublesome symptoms. The enucleation was performed under chloroform anæsthesia. The second case was a son of the previous patient, his mother. This boy, aged 10½ years, had had his tonsils removed at the age of four at the Throat Hospital. At the age of six, after scarlatina, the tonsils again became enlarged and were again removed by St.Clair Thomson with the guillotine. A few months later cheesy collections were noticed in the crypts of the tonsils, and these had continued almost without intermission. The tonsil stumps were deeply embedded between the faucial pillars, riddled with crypts, from some of which dirty-white, fœtid, cheesy matters were easily extruded. The operation in each case was performed chiefly by a pair of curved scissors and the fingers.

**Peritonsilitis.**—Somers[6] has used supra-renal gland in the form of powders, fine grains being placed in his patient's mouth with the instruction to thoroughly masticate it, retaining it as far back in his pharynx as is possible from five to ten minutes, and then cleanse the mouth with hot or cold water, according to the individual peculiarities of the case. Of great importance is the effect of the drug in diminishing and frequently dissipating localised œdema when limited to exposed mucous surfaces. In Somers' hands it has been possible in a few hours to produce complete disappearance of œdema of the uvula, whether due to a localised inflammation or the result of more extensive reaction. He cites two cases in which the result of this treatment was to greatly diminish the pain and swelling, and thus to afford great assistance to the exhibition of the other drugs which are usually employed in these cases.

1 Lancet, April 6, 1901. 2 Med. Record, July 13, 1901. 3 Ibid. 4 Jour. of Laryng., April, 1901. 5 Lancet, Feb. 16, 1901. 6 Merck's Archiv., May, 1901.

